# PD-L1/miR-155 Interplay in Pediatric High-Grade Glioma

**DOI:** 10.3390/brainsci12030324

**Published:** 2022-02-28

**Authors:** Jakub Litak, Wiesława Grajkowska, Jacek Bogucki, Paweł Kowalczyk, Alicja Petniak, Arkadiusz Podkowiński, Justyna Szumiło, Janusz Kocki, Jacek Roliński, Mansur Rahnama-Hezavah, Marcin Roszkowski, Cezary Grochowski

**Affiliations:** 1 Department of Immunology, Medical University of Lublin, 20-093 Lublin, Poland; jakub.litak@gmail.com (J.L.); jacek.rolinski@umlub.pl (J.R.); 2 Department of Pathology, The Children’s Memorial Health Institute, 04-730 Warsaw, Poland; w.grajkowska@ipczd.pl; 3 Chair and Department of Organic Chemistry Medical University of Lublin, 20-400 Lublin, Poland; jacekbogucki@umlub.pl; 4 Department of Neurosurgery, The Children’s Memorial Health Institute, 04-730 Warsaw, Poland; p.kowalczyk@ipczd.pl (P.K.); m.roszkowski@ipczd.pl (M.R.); 5 Department of Clinical Genetics, Medical University of Lublin, 20-090 Lublin, Poland; alicja.petniak@umlub.pl (A.P.); janusz.kocki@umlub.pl (J.K.); 6 Da Vinci Neuroclinic, Tomasza Zana 11A, 20-601 Lublin, Poland; apodkowinski@wp.pl; 7 Department of Clinical Pathomorphology, Medical University of Lublin, 20-090 Lublin, Poland; justyna.szumilo@umlub.pl; 8 Department of Dental Surgery, Medical University of Lublin, 20-081 Lublin, Poland; rahnama.m@interia.pl; 9 Chair of Anatomy, Medical University of Lublin, 20-439 Lublin, Poland

**Keywords:** high grad glioma, pd-L1, miRNA, miR155

## Abstract

High-grade pediatric glioma (p-HGG—WHO 2021, formerly GBM—WHO 2016), as a common, aggressive, and highly lethal primary brain malignancy in adults, accounts for only 3–15% of primary brain tumors in pediatric patients. After leukemia, brain malignancies are the second most common in the pediatric population and first in incidences concerning solid tumors. This study was designed on the basis of 14 pediatric patients hospitalized at Children’s Memorial Health Institute in Warsaw, Poland, due to p-HGG treatment. All the patients had a histopathological diagnosis performed by an experienced neuropathologist according to WHO guidelines (WHO 2016 Grade IV Glioblastoma). A significant correlation was found between the miR-155 concentration and the level of PD-L1 expression in p-HGG tumor tissue. Very few reports have indicated PD-L1 expression in pediatric patients.

## 1. Introduction

Diffuse pediatric-type high-grade glioma (p-HGG—WHO 2021, formerly GBM (glioblastoma)—WHO 2016), as a common, aggressive, and highly lethal primary brain malignancy in adults, accounts for only 3–15% of primary brain tumors in pediatric patients [1]. After leukemia, brain malignancies are the second most common in the pediatric population and first in incidences concerning solid tumors. Among all the pediatric-type diffuse gliomas, which constitute 40–50% of pediatric primary CNS tumors, p-HGGs are not particularly frequent. [1]. Nevertheless, p-HGG presents a high mortality and a significant morbidity equally to the adult population [2]. With a 20% five-year survival, p-HGG represents a disease with extremely poor prognosis. Standard treatment approaches consist of surgery and chemotherapy. Adjuvant radiation is reserved for patients older than 3 years old [3]. Despite treatment, the results are far from ideal. Appropriate modifications of the standard regiment seem inevitable [4]. The epigenetic and molecular aspects of oncogenesis in p-HGG are taken under consideration as a target of more effective therapies [5]. Only 3% of genes contain genetic data necessary in translational processes. Micro RNA molecules (miRNA) are also included, representing 1% of the total human genome [6]. Mostly, miRNAs are located in other genes, especially in exons and introns, controlling their expression and taking part in post-transcriptional regulation. Each molecule controls about 100 genetic trigger points. A complex network of interdependencies regulates cardinal organism functions. The first reports linking miRNAs with brain tumor pathophysiology appeared in the 2000s. Since then, various aspects have been analyzed, and numerous studies have demonstrated the participation of these molecules in the processes of GBM oncogenesis [7]. Several studies have identified miRNAs as indicators of patient survival and have demonstrated their prognostic properties. Among various miRNAs, miR-155 expression was found to be important in many types of tumors, including leukemia, esophageal cancer, prostate cancer, mandibular osteosarcoma, and non-small cell lung cancer [8]. Previous studies have connected miR-155 expression with invasiveness, low apoptosis, and a high proliferation ratio in melanoma and psoriasis patients. Elevated levels of miR-155 in gliomas were associated with unfavorable outcomes. None of these studies found miR-155 in p-HGG [9]. Immune checkpoints (ICs), as trigger points of immune responses, control immune homeostasis and maintain a balance between activation and suppression. PD-L1/PD-1 axis over-signaling inhibits T cells proliferation and promotes regulatory T cells in GBM microenvironments [10]. It promotes GBM cells to immune escape. Some studies have reported the level of PD-L1 expression as a biomarker and linked its expression with poor prognosis. Few reports relate to p-HGG. IC inhibition in pediatric patients has been analyzed, and PD-1/PD-L1 inhibitors such as nivolumab, pembrolizumab, and ipilimumab are observed in ongoing trials [10,11,12]. The present study was performed to correlate miR-155-5p with PD-L1 expression in 14 p-HGG patients. This is the first study that confronts this relation in such a homogeneous group of p-HGG patients. Moreover, we analyze the correlation between miR-155, p53, Olig 2, Ki67, GFAP, and synaptophysin expression.

## 2. Methods

### 2.1. Subjects

This study was designed on the basis of 14 pediatric patients hospitalized at the Children‘s Memorial Health Institute in Warsaw, Poland, due to p-HGG treatment. All patients had a histopathological diagnosis performed by an experienced neuropathologist according to WHO guidelines (WHO 2016 Grade IV Glioblastoma). All patients included in the study received a diagnosis and treatment between 2013 and 2020. Histopathological and clinical data were based on medical records collected during hospitalization at Children‘s Memorial Health Institute Warsaw, Poland. The selection of patients was random, and the main criterion apart from a histopathological diagnosis of GBM was an age below 18 years. All GBM samples were scored for PD-L1 expression and miR-155 concentration as well. The evaluations were carried out by experienced pathologists as well as certified employees of the Genetics Department.

### 2.2. Tumor Samples Collection

The tumor samples were collected during neurosurgical resection from typical parts of the cancer. The collected tissue was preserved with 4% phosphate-buffered formaldehyde. Secondary samples were embedded in paraffin following standard procedure guidelines.

Paraffin sections of 4 μm were H-E-stained according to standard protocols. An autostainer (Dako) was used in the processes of immunohistochemistry after heat-induced epitope retrieval with the participation of citrate buffer.

### 2.3. Evaluation of Immunohistochemistry Findings

A microtome was used to cut paraffin-embedded Pediatric high grade glioma samples into 4 μm sections. Afterward, all slices were put on Thermo Scientific™ SuperFrost Plus Slides (Santa Clara, CA, USA) to improve cell adhesion and perform effective immunohistochemistry. A Dako Omnis IHC platform (GI100, Santa Clara, CA, USA) was used to perform proper analysis. Selected reagents were used in the IHC (immunohostochemistry) analysis: the PD-L1 IHC 22C3 pharmDx staining set (GE006), including a monoclonal PD-L1 antibody 22C3 clone (Dako Omnis, Santa Clara, CA, USA), a negative control (Dako Omnis, Santa Clara, CA, USA), a high pH detective system EnVision Flex Mini Kit (Dako Omnis, Santa Clara, CA, USA, GV823), and a wash buffer (20×) (GC807, Dako Omnis, Santa Clara, CA, USA). In order to remove the paraffin, a low pH Envision Flex Target Retrieval Solution (Dako Omnis, Santa Clara, CA, USA) was used. A positive control was produced using tonsil tissue. A negative control was produced with a Negative Control Reagent. The PD-L1 IHC 22C3 pharmDx staining protocol was used to score PD-L1 expression. The negative control was produced according to the PD-L1 IHC 22C3 pharmDx Negative Control Reagent staining protocol.

### 2.4. Detection of miR-155 Expression in Tumor Samples

Formalin-Fixed Paraffin-Embedded (FFPE) Tumors of Pediatric high-grade glioma were cut using a microtome into 10 μm slices. The Recover All Total Nucleic Acid Isolation Kit for FFPE Tissues (Fisher Scientific, Hampton, NH USA) was used for the total RNA enriched with a miRNA fraction isolation according to the manufacturer’s protocol. The quality of the obtained RNA was determined using micro-capillary electrophoresis using an Agilent 2100 Bioanalyzer (Agilent Technologies, Santa Clara, CA USA) and an Agilent RNA Nano kit (Agilent Technologies, Santa Clara, CA USA). The purity and concentration of RN A obtained from the FFPE was assessed using spectrophotometry (NanoDrop 2000c, ThermoFisher Scientific, Waltham, MA USA). Concentration of hsa-miR-155-5p was determined using enzyme immunoassay for miRNA quantification hsa-miR-155-5p miREIA (BioVendor, Brno, Czech Republic) according to the manufacturer’s protocol. 

### 2.5. Statistical Analysis

Firstly, the quantitative group characterization for parametric variables was presented as standard deviations and means (SD, M). Secondly, frequencies and percentages were used to present parametric values. The study was designed to find correlations between miR-155 expression and PD-L1 expression. Due to non-parametric variables, data were computed with an application of Spearman’s R coefficient. The Mann–Whitney non-parametric U test was performed to verify differences in the subgroups described by the following criteria: supratentorial/infratentorial, tumor occurrence in left/right hemisphere, and male/female. The tests were performed using STATISTICA v.13 (Tibco Corporation, Palo Alto, CA, USA). The results were considered statistically significant at *p* < 0.05.

## 3. Results

The study was based on 14 patients diagnosed with p-HGG. The histopathological diagnosis mean age was 9.64 years old (SD = 6.68), ranging from 3 to 14 years. Ten male pediatric patients and 4 female pediatric patients (71% vs. 29%) were enrolled. Pediatric high-grade glioma was the common diagnosis for all pediatric patients. Every participant was treated with surgery and adjuvant chemo- and radiotherapy (Table 1). Radiological findings were described as a single lesion in all included individuals. A supratentorial presentation of GBM was found in 11 patients, 3 of which had an infratentorial tumor (78% vs. 22%). The right hemisphere was infiltrated in three cases, the left hemisphere in nine cases, and the midline in two cases (21% vs. 64% vs. 15%) (Table 2).

### PD-L1 and miR-155 Expression

**Figure 1:**
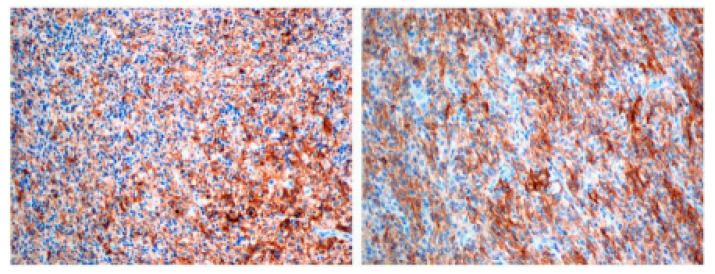
PD-L1 positive staining in p-HGG tumor samples. Magnification 10×.

PD-L1 staining was investigated in tumor cells and were scored as 0 (none), 1 (0–20%), 2 (20–40%), or 3 (>40%). On this scale, PD-L1 was detected in 8 out of 14 patients (57%). Two patients showed third score, three showed second score, and three showed first score (Table 3; Figure 1). In six cases, there was no PD-L1 staining (43%). The mir-155 concentration was evaluated, and the results varied from 0.133 to 2.584 amol/mikrol (Table 3).

**Figure 2:**
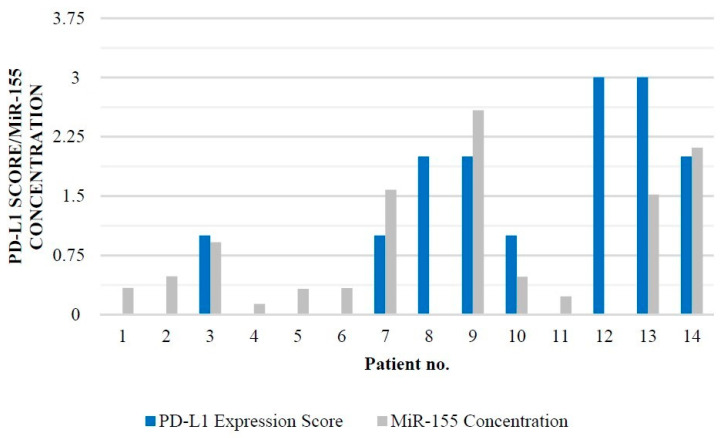
Molecular status of PD-L1 expression score and miR-155 concentration in several patients with p-HGG diagnosis.

When constructing this study, a hypothesis on the correlation between miR-155 concentration hand PD-L1 expression was made. We hypothesized that both parameters could have a strong impact on the general outcome in p-HGG patients. A significant correlation was found between miR-155 concentration and the level of PD-L1 expression in p-HGG tumor tissue (*p* = 0.00087; R = 0.857921; *p <* 0.05) (Figure 2).

**Figure 3:**
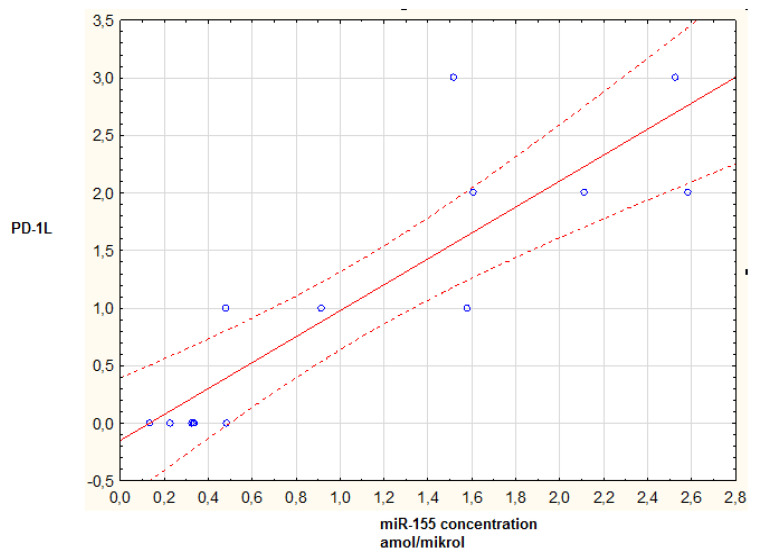
Scatterplot of miR-155 concentration versus PD-L1 expression level. *p* = 0.00087; *p* < 0.05. Blue dot—data, solid red line—regression line, dotted red line—95% CI.

Moreover, there was no significant correlation between the miR-155 concentration and biological parameters such as gender and age (*p* > 0.05, Mann–Whitney U test). Additionally, there was no significant correlation between miR-155 concentration and tumor location in both subgroups (supra/infratentorial and left/mid/right) (*p* > 0.05) (Figure 3). Interestingly, there were also no correlations between miR-155 and Ki67, p53, Olig 2, GFAP, or synaptophysin expression (*p* > 0.05 in all cases).

In sum, our investigation revealed a statistically significant correlation between miR-155 concentration and PD-L1 expression in a homogeneous group of pediatric patients diagnosed with p-HGG.

## 4. Discussion

PD-L1, as an IC molecule, is expressed on the surface of p-HGG cells. Very few reports have indicated PD-L1 expression in pediatric patients. Our study cohort presents 57% of cases (8 out of 14). Similar results were presented by Allison in a 14-case LGG study group with a 50% expression of PD-L1, and Majzner et al. showed 30% in a HGG group [13,14,15]. The expression level of PD-L1 was found as an important factor in predicting the efficacy of ICI-related immunotherapy. The uncertain effectiveness of pembrolizumab, a PD-1 ligand inhibitor, in a small cohort of p-HGG patients led to a study in which unsatisfactory results regarding additional molecular and epigenetic factors were found [16,17]. The present study showed a correlation between PD-L1 expression and the occurrence of miR-155 in a p-HGG cohort. miR-155 is single-stranded and contains a 23-nucleotide miR molecule in the third exon. It is widely featured as an essential factor regulating cardinal pathophysiological processes, including tumor occurrence and development, immune tolerance, inflammation, and hematopoiesis [18,19,20]. miR-155 expression was found in an adult population of high-grade gliomas and correlated with significantly worse outcomes [21]. The strong correlation with glioma grade, invasiveness, migration, and cell proliferation was underlined in a study performed by Wu et al. The blockade of miR-155 limits the growth of pediatric high grade glioma cells in vivo and in vitro. The inhibitory molecule NSC141562 was proposed as an effective blocker of mir-155 expression with promising results [22]. Interestingly, miR-155 occurrence is significantly enhanced by TLR ligands. TLR-4 signaling is a proinflammatory pathway also found in pediatric high-grade glioma and is promoted by miR-155 expression. The miR-155 molecule suppresses inhibitory regulators of inflammation and promotes immune system inefficiency in tumor microenvironments [23]. The correlation between the TLR4 signaling pathway and PD-L1 expression has been well described in previous studies. The miR-155/TLR4/PD-L1/PD-1 axis could be another hypothetical interplay between miR155 and IC [24]. Interestingly, miR-155 expression is responsible for radiotherapy and alkylating agent (Temozolomide) resistance by activation of the nuclear factor (NF)kB in anaplastic gliomas. The methylation of the miR-155 promoter causes the downregulation of this molecule and has been significantly correlated with improved responses to TMZ and a higher survival ratio [24]. In summary, the level of miR-155 methylation is a significant prognostic factor in adult anaplastic gliomas [25]. Another interesting interplay presented by miR-155 is its interaction with IC molecules. The upregulation of mir-155 accelerates pediatric high grade glioma invasion by CTLA-4 and PD-L1 activation. miR-155 interacts with the CD80/CTLA-4 axis, controls the Treg/T-cell ratio in immune responses, and acts as an inhibitory factor of malignant cell elimination [26,27]. Some models of miR-155/PD-L1 interactions have been proposed by Atwa SM et al. A strong interaction between the upregulation of miR-155 and PD-L1 expression in hepatocellular carcinoma was discovered. This model of interplay consists of two steps: miR-155 upregulation and the indirect activation of PD-L1 by long non-coding ribonucleic acid X overexpression. miR-155 can also interact with miR-194 and have a synergistic effect on PD-L1 expression [28]. Peng et al. proved that miR-155 host gene (miR-155HG) expression is associated with IC expression and has a significant prognostic value in multiple cancers [29]. A high expression was related to improved outcomes in lung adenocarcinoma, cholangiocarcinoma, and skin melanoma. Meanwhile, a high expression of miR-155 HG was correlated with unfavorable outcomes in clear cell carcinoma of the kidney, low-grade gliomas, and GBM. Interestingly, high levels of miR-155 are significantly correlated with levels of tissue infiltration by immune cells and the expression of IC molecules, such as CTLA-4 and PD-L1, in most types of malignancies [30,31,32,33,34]. Many studies have reported that the status of IC expression in pediatric high grade glioma microenvironments can predict the efficacy of immunotherapy [35,36]. In our study, we present a statistical correlation between miR-155 and PD-L1 in a small homogeneous group of patients with a rare malignant tumor, namely, p-HGG. The results presented correspond with the thesis that miR-155 has a strong impact on PD-L1 expression and that both are correlated with unfavorable outcomes [37,38]. All previous studies were performed on adult glioblastoma groups of patients or performed on cell lines. It is worth emphasizing that p-HGG has a different genetic profile compared with adult patients and that the course of the disease could deviate from the known model in adults. Despite this, the presented correlation seems to play an important role in the molecular interplay between the epigenetic and immune aspects of p-HGG. Numerous molecules, immune cells, and IC take part in this complicated relation that takes place in glioma microenvironments [39,40].

## 5. Conclusions

We believe that knowledge of every correlation in the complicated network of dependencies can bring us closer to proper molecular profiling, which is useful in modern therapies. Our study has several limitations. The main one is that the results are based on a small study cohort of p-HGG patients. We correlated miR-155 with PD-L1 but excluded other miRNAs which could interfere. p-HGGs very often present various mutations in histone 3 (either H3G35R or H3K27M) resulting in an altered expression through reduced H3 trimethylation. Our further investigation on p-HGGs will by expand by H3mut and H3me status [41]. Our study requires further investigation to find more interesting relationships between IC and miRNA molecules in p-HGG. We believe that molecular and epigenetic profiling could be a golden standard for pediatric patients seeking effective immunotherapy.

## Figures and Tables

**Table 1:** Study group details.

	*n* (%)
Male vs. Female	10 vs. 4 (71% vs. 29%)
Mean age 9.64 years old (SD = 6.68)	
Surgery	14 (100%)
GTR	10 (71%)
PR	4 (29%)
Adjuvant treatment	14 (100%)

**Table 2:** Localization of p-HGG.

	*n* (%)
Single lesion	14 (100%)
Supratentorial	11 (78%)
Infratentorial	3 (22%)
Right	3 (22%)
Left	9 (64%)
Midline	2 (14%)

**Table 3:** Molecular status of PD-L1 expression and miR-155 expression in several patients with p-HGG diagnosis.

Age	Sex	Tumor	PD-L1 Expression Score	MiR-155
		Localization		Amol/Mikrol
14	Male	Left temporal lobe	0	0.337
11	Male	Left frontal lobe	0	0.483
13	Female	Left temporal lobe	1	0.916
12	Female	Left occipital lobe	0	0.133
6	Male	Brainstem	0	0.324
3	Female	Left temporal	0	0.333
10	Male	Right parietal	1	1.579
11	Male	Right temporal	2	1.609
13	Male	Right temporal	2	2.584
5	Male	Left frontal	1	0.479
12	Male	Left cerebellum	0	0.228
11	Male	Left temporal	3	1.609
6	Female	Left frontal	3	1.518
8	Male	Brainstem	2	2.112

## Data Availability

Not applicable.
